# Differences in growth and competition between plants of a naturalized and an invasive population of *Bunias orientalis*

**DOI:** 10.1002/ece3.11153

**Published:** 2024-03-18

**Authors:** Blaise Binama, Müller Caroline

**Affiliations:** ^1^ Department of Chemical Ecology Bielefeld University Bielefeld Germany

**Keywords:** *Bunias orientalis*, competitive ability, interpopulation competition, intrapopulation competition, plant growth

## Abstract

The global shift of species' distributions has led to high numbers of noninvasive naturalized plants and the accumulation of invasive species within ecosystems. Competition between species may influence population dynamics, but little is known about the impacts of competition between conspecifics of naturalized and invasive populations. We investigated several plant traits at initial growth and regrowth following artificial defoliation in intra and interpopulation competition. Therefore, we used plants of *Bunias orientalis* from one noninvasive naturalized and one invasive population grown alone or in competition of two or three. Plants from the naturalized population were expected to be less competitive than plants from the invasive population, reflecting their differential impact in the introduced range. Independent of status, intrapopulation competition was expected to have less negative impacts on plants than interpopulation competition. Our results show that competition impacted mostly growth‐ rather than physiology‐related traits. The relative magnitude of intra and interpopulation competition differed among plant traits at the first and second harvest. Plants of the invasive population outperformed the naturalized population by allocating relatively more resources to the aboveground biomass and producing more and longer leaves particularly when grown in competition against two plants. Moreover, plants of the invasive population were more competitive, which may influence their successful establishment and range expansion in the introduced range, but growth patterns differed after artificial defoliation. Although evolution of intrapopulation competition in naturalized and invasive ranges may be expected, interpopulation competition seems to adversely impact the performance of the naturalized plant population of *B. orientalis* studied here. Apart from the status (naturalized vs. invasive), other factors may have had an influence on plant performance. Thus, further research is needed with more naturalized and invasive populations to test the generality of our findings and to isolate the specific mechanisms driving differences in competitiveness.

## Graphical abstract

We studied how a naturalized and an invasive population of *Bunias orientalis* differ when (re)‐grown alone, in intra and in interpopulation competition. There was no difference in aboveground biomass between the two populations, but plants of the invasive population produced more and larger leaves and showed a stronger competitive ability, which may enhance the ability of *B. orientalis* to establish and expand in its invading range. The findings presented here provide insight into how the complex interactions between plants from different ranges may influence population dynamics.

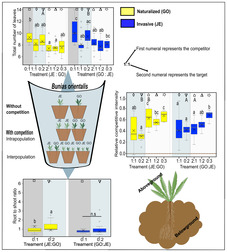

## 1 INTRODUCTION

The increase in the number of alien plant species (Seebens et al., 2017; van Kleunen et al., 2015) has triggered research on factors that contribute to successful invasions (van Kleunen et al., 2018; Zhang et al., 2019). The position of alien species along the introduction–naturalization–invasion continuum is thereby a fundamental aspect that should be considered in trait‐based studies of invasiveness (Blackburn et al., 2011; Richardson et al., 2000). In particular, plants of noninvasive naturalized populations, which reproduce in the wild but do not expand rapidly in their introduced range, may exhibit different characteristics than plants of invasive populations, which reach high abundances in the invaded ecosystems and have substantial ecological or economic impacts (Richardson & Pyšek, 2012), due to contrasting selection pressures in their respective ranges. Yet, our knowledge about the growth and competition between plants of naturalized and invasive populations remains limited. In contrast, several studies have focused on competition between plants of native and invasive populations (Bossdorf et al., 2004; Vilà et al., 2003; Zheng et al., 2015). It is usually postulated that plants from invasive ranges are more competitive than their conspecifics from native ranges (Garcia‐Serrano et al., 2007; Golivets & Wallin, 2018; Kuebbing & Nuñez, 2016). The competitive abilities and ecological impacts of invasive plants prove to be far more pronounced in their non‐native ranges, visible in significantly higher population densities and individual growth potential than in their native ranges (Callaway et al., 2012; Parker et al., 2013).

The competitive ability of invasive plants has received much attention (Levine et al., 2003; Sheppard, 2019; Sheppard & Brendel, 2021). The evolution of increased competitive ability (EICA) hypothesis postulates that introduced plants are no longer under the control of natural enemies. Therefore, they may reduce costly defense traits, releasing resources and energy that can be reallocated from defense to growth (Blossey & Notzold, 1995). So far, support for the EICA has been inconclusive (Müller, 2018). In favor of EICA, individuals of several species such as *Lythrum salicaria*, *Senecio jacobaea*, and *Sapium sebiriferum* from the introduced ranges were larger than conspecifics from the native range, but seemed less resistant towards herbivores (Blossey et al., 1996; Blossey & Notzold, 1995; Lin et al., 2018; Siemann & Rogers, 2001). Conversely, other studies have found no evidence for EICA. For example, the competitive ability of plants from invasive populations of *Hypericum perforatum* did not differ from that of plants from native populations (Vilà et al., 2003), while plants from invasive populations of *Alliaria petiolata* had even a lower competitive ability than plants from native populations (Bossdorf et al., 2004). Thus, the species identity of the competitors but also the test populations may have an impact on the outcomes of competition experiments (Qin et al., 2013).

Plant competition should be determined by investigating both intraspecific and interspecific competition because both affect the distribution pattern of non‐native plants in their introduced range (Bossdorf et al., 2004; Gioria & Osborne, 2014; Vilà & Weiner, 2004). Interspecific competition is considered as an important factor driving plant invasions (Crawley et al., 1990; Sheppard, 2019), but competitive ability can also differ significantly within species among populations (Huang & Peng, 2016; Lin et al., 2015), as shown, for example, for different invasive populations of *Taraxacum officinale* and *Ageratina adenophora* or native populations of *T. platycarpum* (Lee et al., 2021; Zheng et al., 2015). Young founder populations exploiting new habitats may initially be less competitive. Once populations are longer established, intraspecific competition may increase, exerting strong selection pressure (Lankau & Strauss, 2011; Wang et al., 2015). Thus, plants of the same species from noninvasive naturalized and invasive populations may differ in their inter‐ or intrapopulation competitive abilities. Moreover, intrapopulation competition may have less impact on plants than interpopulation competition, independent of population status.

Many invasive species thrive in disturbed or unstable habitats, like those caused by regular mowing or soil perturbation, or in early succession sites (Dietz et al., 1999; Meyer et al., 2021). Early in succession, when soil nitrogen (N) and organic matter levels are low, there is competition for the limited resources and plants may quickly acquire resources while preventing neighbor plants from doing the same (Song et al., 2017). Plant traits such as a high seed germination rate (Ferreras et al., 2015), rapid initial growth with high nutrient acquisition and high morphological plasticity may enhance the re‐establishment of populations after habitat disturbance (Dietz et al., 1999; Funk, 2013). Hence, plants with quick growth and effective regrowth capacities may thus be quite successful. Various plant species store reserves such as carbohydrates belowground in the roots (Donaghy & Fulkerson, 1997; Lin et al., 2018; McCormick et al., 2013). Utilizing these stored reserves enables them to sustain tissue loss due to defoliation and be able to regrow (Lin et al., 2018; McNaughton, 1983). Thus, competitive ability should be determined not only during initial growth but also during regrowth periods. Storing resources for regrowth can be traded off with growth (van der Meijden et al., 1988). While it is well documented that many invasives thrive in disturbed habitats, other studies suggest a potential trade‐off between initial growth and regrowth capacity, as found, for example, in *Jacobaea vulgaris* (Lin et al., 2018). Such trade‐offs, although not extensively tested, may contribute to variation in regrowth abilities among populations.

*Bunias orientalis* L. (Turkish rocket, Brassicaceae) is a suitable candidate to study inter‐ versus intrapopulation competitive growth as it can have native, naturalized or invasive status in different areas. Plants from different populations have been shown to differ in various growth‐ and defense‐related traits such as total leaf number and leaf mass per area (marginally significant) when grown under common garden conditions without competition (Binama & Müller, 2022; Tewes & Müller, 2018), but little is known how they perform in competition. The lanceolate leaves rapidly expand in length rather than width (pers. observation). This characteristic has been linked to the ability of plants of different species to effectively capture light resources, with longer leaves being associated with a higher specific leaf area (Liu et al., 2016; Pérez‐Harguindeguy et al., 2013). Moreover, *B. orientalis* plants are known to regrow well from root fragments, supporting their rapid establishment at disturbed sites (Birnbaum, 2006). Thus, in the present study, we investigated the growth and competitive ability of a naturalized and an invasive population of *B. orientalis* when plants of these grew alone, with one or two neighbors of the same population, i.e. in intrapopulation competition, or with one or two neighbors of another population, i.e. in interpopulation competition. Because aboveground competition may have a stronger influence on species dominance than belowground or competition in both compartments (Kiær et al., 2013), we mainly focused on aboveground plant traits. We hypothesized that (1) plants of the invasive population produce more biomass as well as more and longer leaves when grow alone than in inter‐ or intrapopulation competition of two or three plants and (2) plants of the invasive population are more competitive than plants of the naturalized population. (3) Independent of population status, intrapopulation competition was expected to have less impact on plants, i.e. reduce biomass less, than interpopulation competition. Finally, (4) plants of the invasive population were expected to show a reduced regrowth capacity because storing carbohydrate resources for regrowth may trade off with initial growth prior to defoliation. Other (co‐founding) factors apart from invasion status may have also differed between these two populations. Thus, we cannot exclude that trait differences may be assigned to such factors not investigated here.

## 2 MATERIALS AND METHODS

### 2.1 Plant material

The species *B. orientalis* is native to South‐East Europe and Western Asia, from where it spread in the 1600s across the Caucasus to Central Europe (Birnbaum, 2006; Koch et al., 2017), where it reached invasive status in parts of Central Europe (Birnbaum, 2006; Dietz et al., 1999). In other parts of Western and Northern Europe, *B. orientalis* is considered naturalized (Harvey et al., 2010; Tewes & Müller, 2018). The term “naturalized” describes populations that have established and sustained themselves outside their native range, with or without human intervention, over numerous life cycles (Richardson et al., 2000). For the experiment we used seeds originating from two populations, one considered as naturalized in Gondreville, France (GO_NA) (latitude: 48°41.23′ N, longitude: 5°57.9′ E) and one considered as invasive in Jena, Germany (JE_IN) (latitude: 50°52.42′ N, longitude: 11°35.76′ E; also used in Tewes & Müller, 2018). In Gondreville, plants of *B. orientalis* are quite abundant but the population has not expanded quickly or became dominant. In contrast, in Jena, the plants can form dense stands and populations quickly expanded as a noxious invader. For the experiment, plants were grown from seeds collected at these sites under standardized conditions in Bielefeld (Germany) ensuring only within population cross‐fertilization (Tewes & Müller, 2018), and seeds of the F1 populations were obtained at least from four mother plants and used for the present experiment to reduce maternal effects.

### 2.2 Study design

**FIGURE 1:**
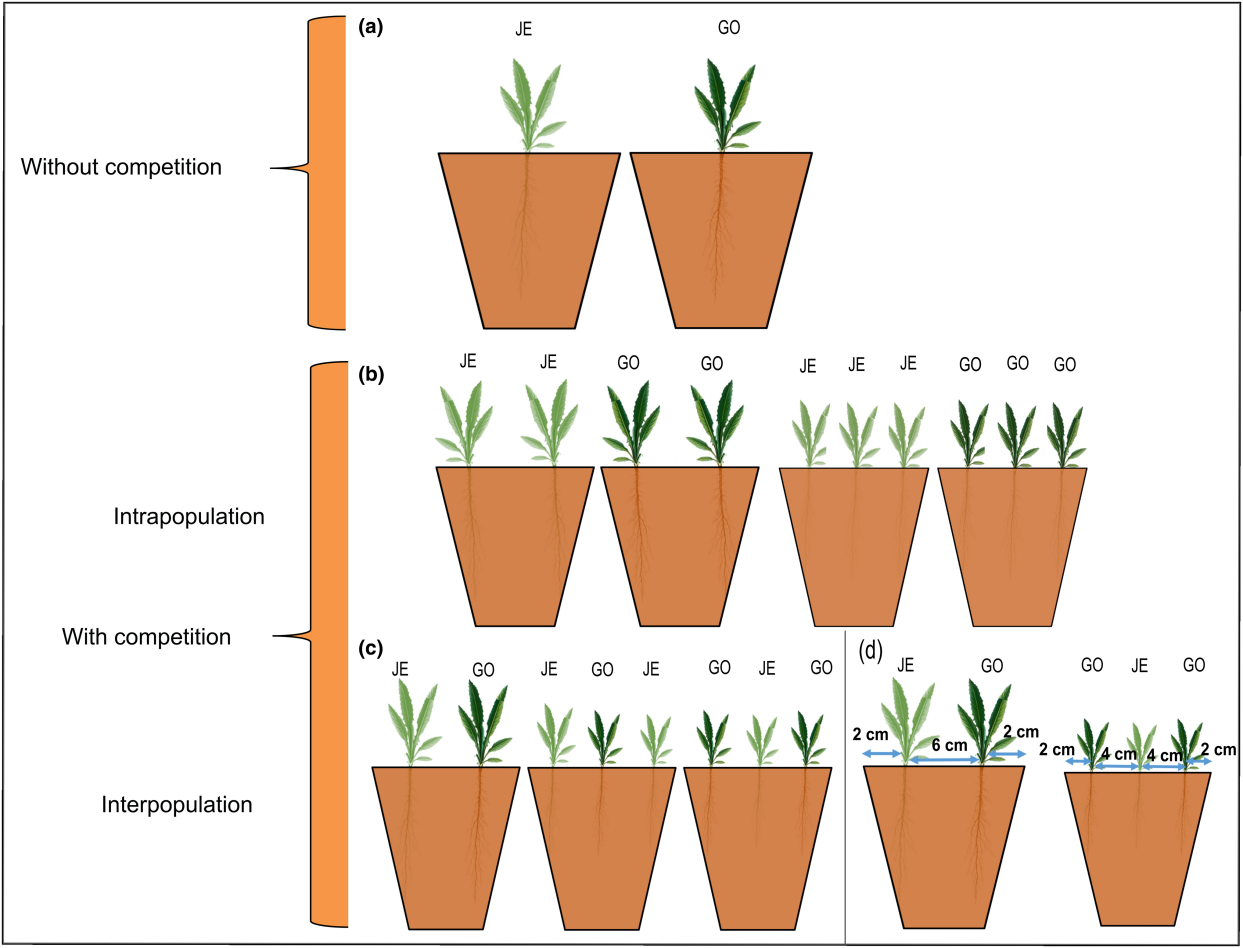
Design of experimental set‐up. *Bunias orientalis* plants from a naturalized (GO) and an invasive (JE) population were grown in 2 L pots in a replacement series; (a) plants grown alone, (b) plants grown in competition of two or three (intrapopulation competition), (c) plants grown in competition of two or three (interpopulation competition). In (d), the initial distance between the seedlings is given at which the two or three individuals of similar size were placed together in the same pot.

In August 2022, silicles of the two populations were cracked, sterilized and germinated following the method described earlier (Binama & Müller, 2022). After six days of growth in the dark, 132 seedlings per population were individually weighed to determine their initial biomass and then transferred in 2 L pots (14 × 14 × 14.5 cm) filled with poorly fertilized soil (C 710 with Cocopor, Stender, Schermbeck, Germany) in different combinations, using a replacement series. The nomenclature of the replacement series introduced by Paulus and Marshall (2022) was used, whereby the first numeral represents the competitor and the second represents the target population (e.g., 2:1 with GO_NA as the competitor and JE_IN as the target would include two individuals of the GO_NA and one individual of the JE_IN population). Plants were grown either alone, used as a control (0:1, containing only one individual of the target population), in interpopulation competition (1:1, 2:1, 1:2), or in intrapopulation competition (0:2, containing two individuals of the target population or 0:3, containing three individuals of the target population). For each treatment, 15 pots were set up, except for the intrapopulation competition 0:3, for which only 6 (GO_NA) and 7 (JE_IN) pots were set up, respectively. Thus, in total we had 263 plants in a total of 118 pots. After a first harvest applied as artificial defoliation, plants from 2 pots of GO_NA (1:1) and 1 pot of JE_IN (0:1) did not regrow well. For combinations in which two or three individuals were placed in the same pot, seedlings of matching size were placed together at a distance of approximately 6 cm or 4 cm along the diagonal, respectively (Figure 1). All pots with plants were arranged randomly in a controlled growth chamber (20°C; 16 h:8 h, light:dark photoperiod; 60% relative humidity) under Osram Fluora lamps (L 36W/77; Osram, Munich, Germany) and were watered three times per week. The position of the pots was changed randomly whenever plants were watered. After 2 weeks of growth in the pots, all plants were fertilized with 1 mL/L Wuxal Super (8‐8‐6 NPK, Aglukon Spezialdünger, Düsseldorf, Germany). When plants were planted alone, in competition of two, or in competition of three, 20, 40, and 60 mL of nutrient solution were added to each pot once per week, respectively. Fertilizer amounts were adjusted to ensure a prolonged growing according to the number of plants in each pot; these amounts had no influence on the observed differences in plant traits.

### 2.3 Data collection and measurements

After 6 weeks of growth, a first harvest (artificial defoliation) was performed. For each plant individual, the numbers of leaves and the length of the longest leaf were directly measured. This species has lanceolate leaves with irregularly sawed leaf margins. Thus, measuring leaf length is straightforward, and leaf length may be a good indicator of plant growth (Steinlein et al., 1996). Moreover, from plants of the replacement series 0:1, 1:1 and 0:2 only, the specific leaf area and the carbon and nitrogen content were determined; therefore, eight leaf disks (10 mm diameter) were cut from one leaf of the second‐youngest, fully developed leaf pair using a cork borer, weighed, and placed in a 2 mL Eppendorf tube. The leaf material was immediately frozen in liquid nitrogen, stored at −80°C until freeze‐drying for 24 h and weighed again to obtain the specific leaf area, calculated by the ratio of leaf area to leaf dry mass. The same leaf material was then homogenized and about 2 (± 0.1) mg used to determine the carbon and nitrogen content, using a C/N‐analyzer (Vario MICRO cube, Elementar, Hanau, Germany). The remaining aboveground biomass was cut from all plants approximately at 2 cm above the soil, weighed, placed in a paper envelope, dried at 50°C for 4 days in an oven and then weighed again to determine the dry biomass and to calculate the water content of the fresh material. For data standardization in replacement series with two or three individuals in one pot, the values were scaled to a single plant per population and pot by dividing the values by the respective number of plants of one population initially grown in each pot.

After the first harvest all plants were allowed to regrow for another 6 weeks under the same growth conditions. After 6 weeks, all individuals were harvested for a second time (“second harvest”) and all aboveground traits were re‐measured. In addition, the main and secondary roots were harvested, dried and weighed and the root to shoot ratio was determined as the ratio of root to aboveground dry biomass. Roots were only collected from plants grown alone or in intrapopulation competition but not in interpopulation competition, because the roots of the latter could not be assigned to the different individuals. We calculated the relative competitive intensity (RCI) in two ways, relative to the performance of control plants or relative to plants grown in intrapopulation competition, as in Williams and McCarthy (2001), with biomass as the measure of yield, i.e., (biomass_cont_ − biomass_comp_)/biomass_cont_, where biomass_cont_ is the average of the aboveground biomass of plants grown in a control and biomass_comp_ is the aboveground biomass of plants grown in inter‐ or intrapopulation competition, as well as (biomass_intra_ − biomass_inter_)/biomass_intra_, where biomass_intra_ is the average of the aboveground biomass of plants grown in intrapopulation competition and biomass_inter_ is the aboveground biomass of plants grown in interpopulation competition, respectively. For the RCI, positive values indicate a competitive impact of the competitor on the target individual, whereas negative values indicate a positive impact on the target.

### 2.4 Statistical analyses

All analyses were performed using R statistical software, version 4.2.2 (R Core Team, 2022). Comparisons between populations for aboveground biomass, total leaves number, length of the longest leaf, carbon and nitrogen content, leaf mass per area, leaf water content, and relative competition intensity were performed using linear mixed‐effect models (LMMs) with the lmer function and lme4 R‐package (Bates et al., 2015). The DHARMA package was used to test the best fitted vs. residual models (Hartig, 2020). As a first step, an “overall” comparison of individual traits for plants grown either in competition of two or three plants was done between the two populations regardless of replacement series. For this, population was treated as a fixed factor, while initial seedling biomass was used as a covariate and replacement series were included as random effects. The LMMs were fitted with a maximum likelihood approach, and *p* values were obtained using a type III Anova with Chi‐square tests. In the second step, all traits were compared within the replacement series for each target population separately, avoiding multicollinearity issues. This involved testing traits of plants of the JE_IN population as target in competition with those from the GO_NA population, or GO_NA as target in competition with JE_IN, using a simple linear model (LM) with lm function (lower‐case letters in figures). Here, replacement series was considered as fixed factor, whereas initial seedling biomass was included as a covariate. The Shapiro–Wilk test and the Levene test (Fox & Weisberg, 2019) were used to test for data distribution and homoscedasticity. The data were log or square root transformed as needed to fulfill the requirements of a normal residual distribution for both LMMs and LMs (see Tables 1, 2). The *p* values for all LMs were evaluated using *F* tests. When statistically significant differences were found, Dunnett's test (Tallarida & Murray, 1987) was used as a post‐hoc test to compare each trait of the target population in interpopulation (1:1, 2:1, 1:2) and intrapopulation (0:2, 0:3) competition to the control trait (0:1), following prior studies on competition experiments (Chen et al., 2021; Zhang et al., 2019). In plants showing significantly longer leaves, we tested for a correlation between the length of the longest leaf and specific leaf area using Spearman rank correlation. Finally, all traits, including root to shoot ratio and root biomass, were compared between plants grown in control, in intra‐ and interpopulation competition, in which the number of neighboring plants was the same, i.e. within control plants (0:1), within competition of two (1:1, 0:2) and within competition of three (2:1, 1:2, 0:3) separately (identical symbols with upper‐case letters in figures). Similar to the second step, simple LMs were used. Population was treated as a fixed factor, while initial seedling biomass was treated as a covariate. All raw data is provided in Table S1.

**TABLE 1:** Overall differences in traits between *Bunias orientalis* plants of the naturalized population GO and plants of the invasive population JE when grown alone, in interpopulation competition or in intrapopulation competition.

Traits compared the overall between populations	Population in competition of two						Population in competition of three					
	First harvest			Second harvest			First harvest			Second harvest		
	*χ* ^2^	(*df*)	*p*	*χ* ^2^	(*df*)	*p*	*χ* ^2^	(*df*)	*p*	*χ* ^2^	(*df*)	*p*
Aboveground biomass (g)	1.34	(1)	.247	0.00	(1)	.974	1.44	(1)	.23	9.14	(1)	**.002** ^**a**^
Total number of leaves	0.53	(1)	.468^a^	5.47	(1)	**.019**	3.5	(1)	.061	8.11	(1)	**.004**
Length of the longest leaf	0.00	(1)	.968^a^	1.82	(1)	.178	6.07	(1)	**.014**	4.09	(1)	**.043**
Specific leaf area (mm^2^ mg^−1^)	2.93	(1)	.087	0.05	(1)	.829^a^						
Nitrogen content (%)	1.54	(1)	.215^b^	0.00	(1)	.954^b^						
Carbon content (%)	1.71	(1)	.192^b^	12.24	(1)	**≤.001**						
Carbon to nitrogen ratio (%)	0.00	(1)	.976	0.36	(1)	.547^b^						
Water content (%)	9.71	(1)	**.002**	0.29	(1)	.593	1.19	(1)	.276	7.85	(1)	**.005**
RCI (control)	3.23	(1)	.072	4.63	(1)	**≤.001**	9.55	(1)	**.002**	3.16	(1)	.076
RCI (intrapopulation)	0.32	(1)	.579	1.31	(1)	.263	7.36	(1)	**.007**	4.72	(1)	**.029**
Root‐to‐shoot ratio (g)				3.61	(1)	.068^b^						
Root biomass (g)				6.46	(1)	**.017** ^**b**^						

*Note*: Traits were analyzed using LMMs. The maximum likelihood method was used to fit all of the models, and likelihood ratio tests (chi‐square tests) were used to determine the *p* values; *df* in parentheses: degrees of freedom. Significant values (*p* ≤ .05) are highlighted in bold. The RCI was calculated relative to performance of plants grown in control or in intrapopulation competition. The letters “a” and “b” represent traits that were transformed by square root or log transformation, respectively.

**TABLE 2:** Differences in traits between plants of *Bunias orientalis* growing in different replacement series of the naturalized population GO and invasive population JE when grown alone, in interpopulation competition or in intrapopulation competition.

Traits compared between replacement series	First harvest						Second harvest					
	GO population			JE population			GO population			JE population		
	*F*	(*df*)	*p*	*F*	(*df*)	*p*	*F*	(*df*)	*p*	*F*	(*df*)	*p*
Aboveground biomass (g)	21.62	(5)	**≤.001**	15.84	(5)	**≤.001** ^**a**^	8.57	(5)	**≤.001** ^**a**^	4.86	(5)	**≤.001** ^**a**^
Total number of leaves	4.67	(5)	**≤.001** ^**b**^	5.38	(5)	**≤.001** ^**b**^	23.76	(5)	**≤.001** ^**b**^	2.09	(5)	.075^a^
Length of the longest leaf	13.68	(5)	**≤.001** ^**a**^	10.58	(5)	**≤.001**	3.92	(5)	**.006** ^**b**^	3.04	(5)	**.023** ^**a**^
Specific leaf area (mm^2^ mg^−1^)	0.54	(2)	.585^b^	5.45	(2)	**.008**	0.05	(2)	.954	2.29	(2)	.115
Nitrogen content (%)	0.37	(2)	.693^b^	2.09	(2)	.137^b^	0.33	(2)	.721^b^	6.65	(2)	**.003** ^**a**^
Carbon content (%)	2.45	(2)	.125^b^	0.98	(2)	.385^b^	1.33	(2)	.257	0.44	(2)	.648^b^
Carbon‐to‐nitrogen ratio (%)	0.37	(2)	.693^b^	8.50	(2)	**≤.001** ^**b**^	1.22	(2)	.307	5.82	(2)	**.006**
Water content (%)	0.33	(5)	.896	3.32	(5)	**.009** ^**a**^	2.32	(5)	.066	0.89	(5)	.487
RCI (control)	10.52	(4)	**≤.001**	3.47	(4)	**.013** ^**a**^	39.27	(4)	**≤.001** ^**a**^	0.79	(4)	.537
RCI (intrapopulation, 0:3)	2.24	(1)	.146	4.21	(1)	**.049**	0.16	(1)	.692	2.53	(1)	.123
Root‐to‐shoot ratio (g)							4.96	(1)	**.036** ^**b**^	2.23	(1)	.148^b^
Root biomass (g)							3.96	(1)	.057^a^	9.20	(1)	**.005** ^**b**^

*Note*: Traits were analyzed using simple linear models. The *p* values were determined using *F* tests; *df* in parentheses: degrees of freedom. Significant values (*p* ≤ .05) are highlighted in bold. Populations in the first harvest *n* = 15, populations in the second harvest *n* = 13–15, RCI was calculated relative to performance of plants grown in control or in intrapopulation competition. The letters “a” and “b” represent traits that were transformed by square root or log transformation, respectively.

## 3 RESULTS

**FIGURE 2:**
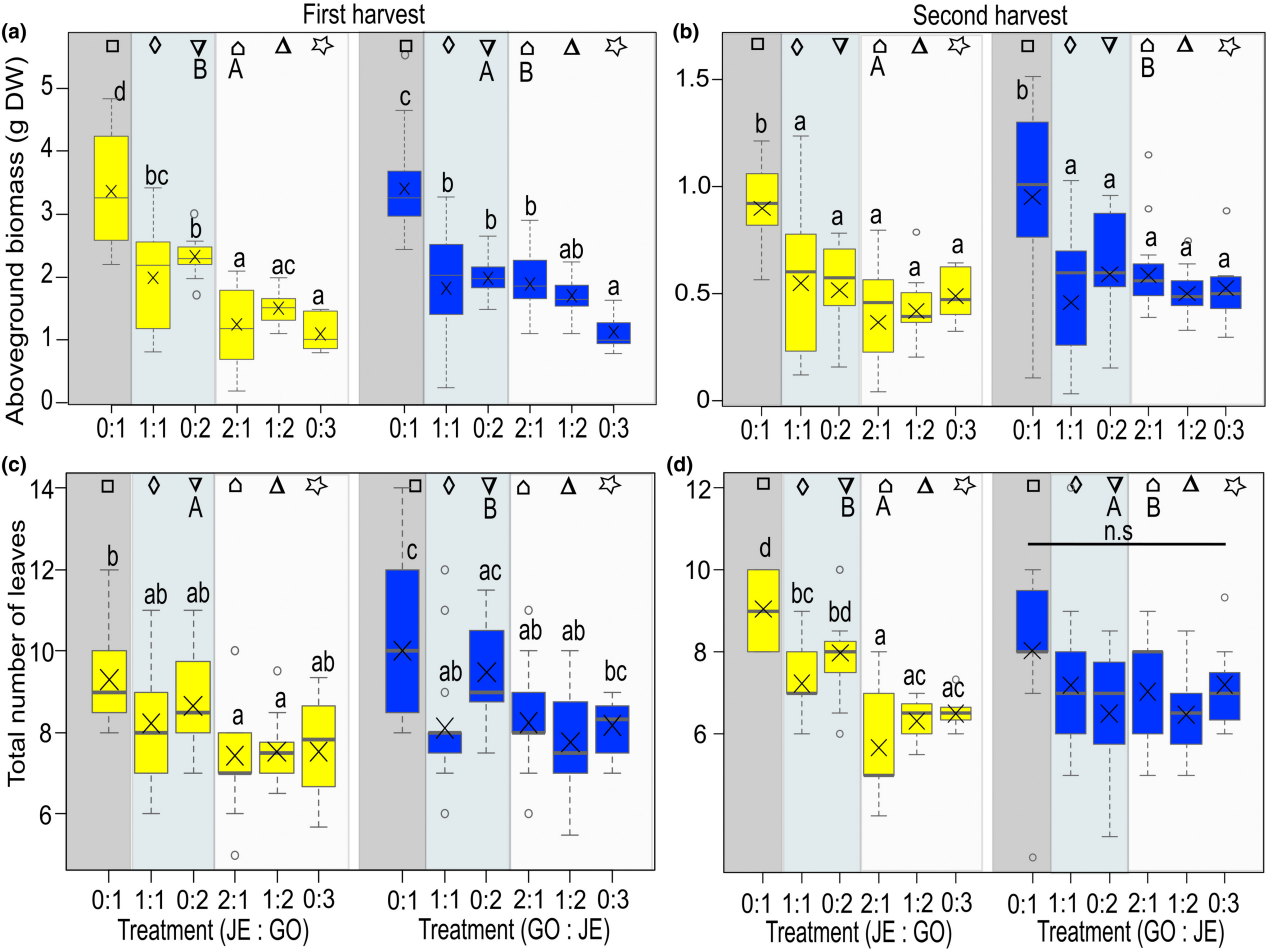
Growth traits of *Bunias orientalis* from plants of the naturalized population GO (yellow) and plants of the invasive population JE (blue), represented by (a, b) aboveground biomass at the first harvest (artificial defoliation; a) and second harvest (after regrowth; b) and (c, d) total number of leaves at the first (c) and second (d) harvest when grown in a replacement series: alone, in interpopulation competition or in intrapopulation competition (number competitor: number target). Data are presented as box whisker plots, boxes represent the 25th and 75th percentiles and medians, crosses show means, whiskers mark minimum and maximum within 1.5‐fold interquartile ranges and open dots are outliers, *n* = 13–15. The shaded boxes represent the competition treatments (*n* = 13–15 for control, *n* = 29–30 for competition of two and *n* = 36–37 for competition of three). Letters indicate significant differences between replacement series (lower‐case letters) according to Tukey posthoc tests, n.s. indicates no significant difference (*p* > .05). Identical symbols indicate pairwise comparisons between plants of both populations grown under similar conditions, with different capital letters representing a significant difference between populations (*p* ≤ .05).

**FIGURE 3:**
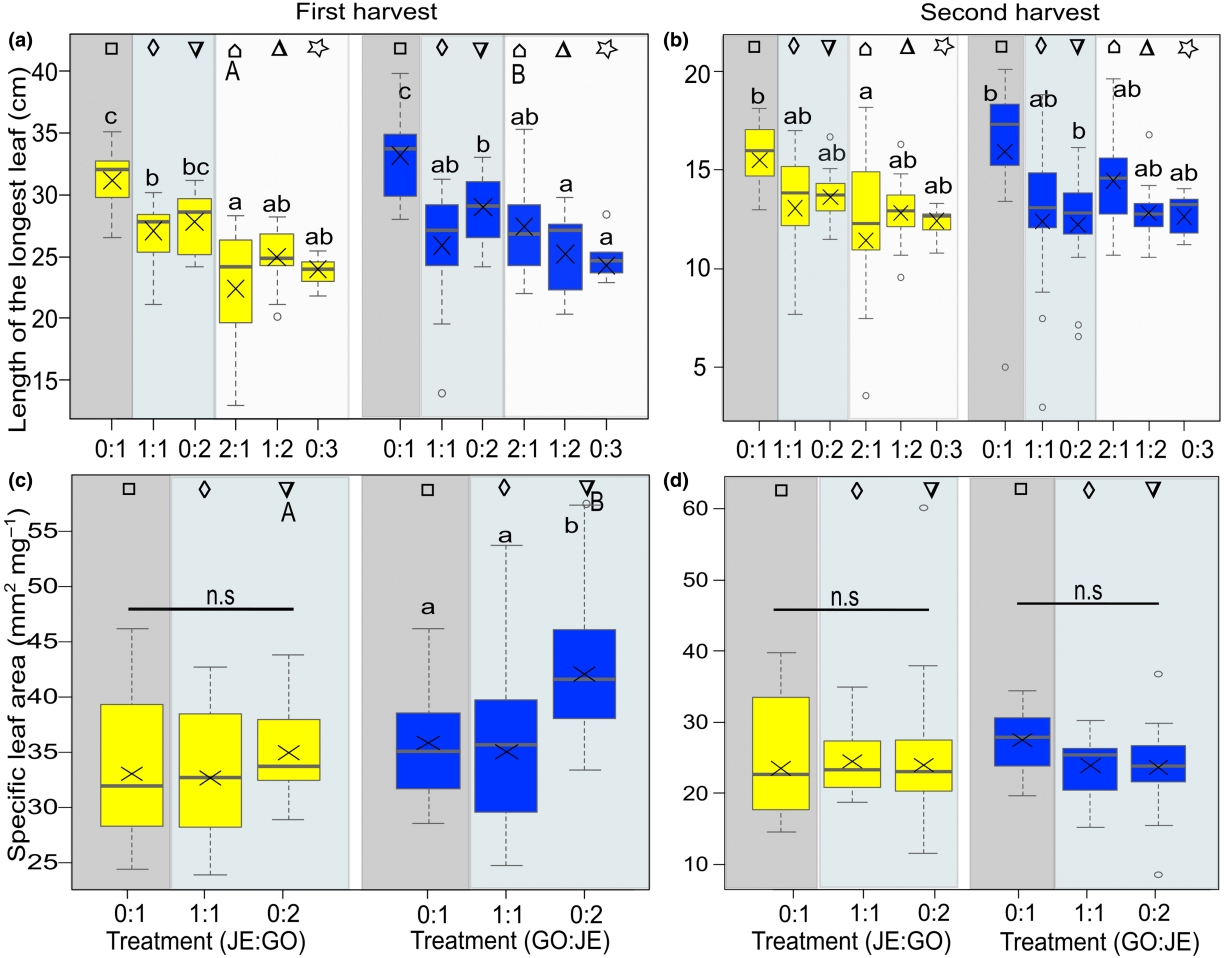
Growth traits of *Bunias orientalis* from plants of the naturalized population GO (yellow) and plants of the invasive population JE (blue), represented by (a, b) length of the longest leaf at the first harvest (artificial defoliation; a) and second harvest (after regrowth; b) and (c, d) specific leaf area at the first (c) and second (d) harvest when grown in a replacement series: alone, in interpopulation competition or in intrapopulation competition (number competitor: number target). Description of box and whisker symbols follow Figure 2, *n* = 13–15 for replacement series. The shaded boxes represent the competition treatments (*n* = 13–15 for control, *n* = 29–30 for competition of two and *n* = 36–37 for competition of three). Letters indicate significant differences between replacement series (lower‐case letters) according to Tukey posthoc tests, n.s. indicates no significant difference (*p* > .05). Identical symbols indicate pairwise comparisons between plants of both populations grown under similar conditions, with different capital letters representing a significant difference between populations (*p* ≤ .05).

**FIGURE 4:**
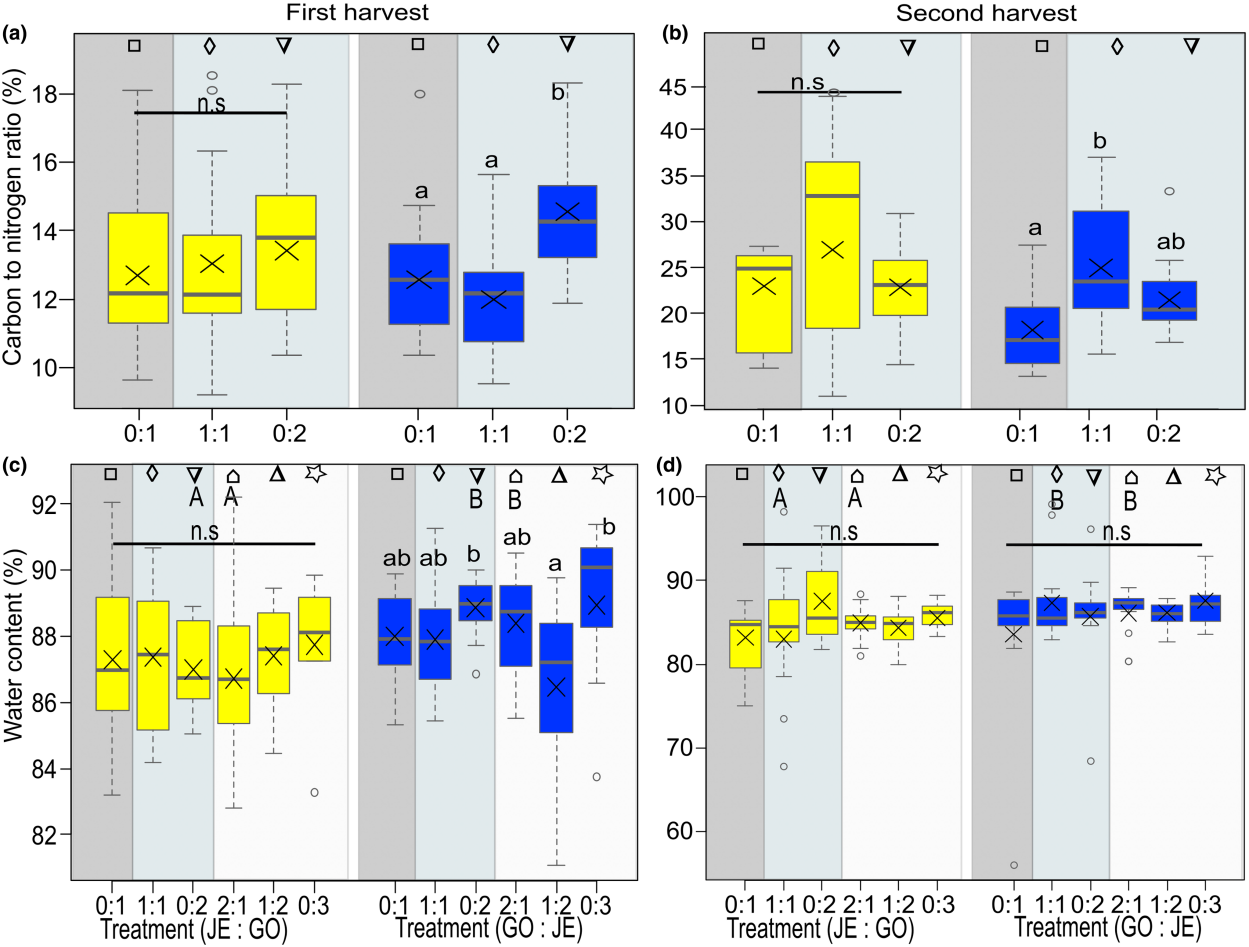
Physiological traits of *Bunias orientalis* from plants of the naturalized population GO (yellow) and plants of the invasive population JE (blue), represented by (a, b) carbon to nitrogen ratio at the first harvest (artificial defoliation; a) and second harvest (after regrowth; b) and (c, d) water content at the first (c) and second (d) harvest when grown in a replacement series: alone, in interpopulation competition, or in intrapopulation competition (number competitor: number target). Description of box and whisker symbols follow Figure 2, *n* = 13–15 for replacement series. The shaded boxes represent the competition treatments (*n* = 13–15 for control, *n* = 29–30 for competition of two and *n* = 36–37 for competition of three). Letters indicate significant differences between replacement series (lower‐case letters) according to Tukey posthoc tests, n.s. indicates no significant difference (*p* > .05). Identical symbols indicate pairwise comparisons between plants of both populations grown under similar conditions, with different capital letters representing a significant difference between populations (*p* ≤ .05).

Overall, plants of the GO_NA (naturalized) and JE_IN population (invasive) of *B. orientalis* did not differ in aboveground biomass when plants were grown in competition of two individuals (1:1, 0:2), neither at the first harvest nor the second harvest, i.e. before and after re‐growth (Figure S1a, Table 1). However, when plants were grown in competition of three individuals (2:1, 1:2, 0:3), there was an overall significant difference between populations at second harvest, with plants of GO_NA having a lower aboveground biomass compared to JE_IN plants (Figure S1a, Table 1). Moreover, there were significant differences between the replacement series for plants of the GO_NA and JE_IN population at both the first and second harvest (lower‐case letters, Figure 2a,b, Table 2); both intra‐ and interpopulation competition resulted in a reduced biomass compared to plants grown alone (control, 0:1). In addition, there were significant differences among GO_NA and JE_IN plants growing in different replacement series for the aboveground biomass at first harvest. Specifically, for GO_NA plants, those grown in intra and interpopulation competition with three plants (2:1, 1:2, and 0:3) had a lower biomass than those grown in competition with two plants (1:1 and 0:2). On the other hand, among JE_IN plants, only those grown in intrapopulation competition (0:3) showed a reduced biomass compared to those grown in the replacement series 0:2 and 2:1 (Figure 2a). Pairwise comparisons between GO_NA and JE_IN plants growing under comparable conditions indicated only a few differences in the measured traits (upper‐case letters, Figures 2, 3, 4). Notably, when grown in intrapopulation pairs, plants of GO_NA had a higher aboveground biomass than plants of JE_IN at the first harvest (Figure 2a). In contrast, in interpopulation competition of three plants (2:1), plants of JE_IN had a higher biomass than plants of GO_NA at both the first and second harvests (Figure 2a,b).

The total number of leaves showed a significant overall difference between populations only at the second harvest; when grown in competition of two individuals, GO_NA plants exhibited more leaves than JE_IN plants. However, when grown in competition of three plants, GO_NA plants had overall fewer leaves than JE_IN plants (Figure S1b, Table 1).

In contrast to the total number of leaves, the length of the longest leaf differed significantly between populations at the first and second harvests, with JE_IN plants having longer leaves than GO_NA plants, but only when grown in competition of three plants (Figure S1c,d, Table 1). In addition, there were significant differences in all replacement series at both the first and second harvests; competition with either GO_NA or JE_IN resulted in a shorter leaf length compared to control growth (0:1) (lower‐case letters, Figure 3a,b, Table 2). For JE_IN as the target, leaf length was significantly shorter when plants were grown in intrapopulation competition (0:2) than when grown alone (0:1) (Figure 3b). Under interpopulation competition (2:1), differences between populations were found, with GO_NA plants having shorter leaves than JE_IN plants at the first harvest (Figure 3a).

Specific leaf area did overall not significantly differ between populations when plants were grown in competition of two plants (Table 1). However, with JE_IN as target, the specific leaf area differed between plants of the different replacement series at first harvest, with plants grown in intrapopulation competition having a higher specific leaf area than control plants or plants grown in interpopulation competition (lower‐case letters, Figure 3c, Table 2). In contrast, with GO_NA as target at the first harvest and for all data at the second harvest, specific leaf area did not differ between plants (Figure 3c,d). In the context of intrapopulation competition at the first harvest, JE_IN plants showed a significantly higher specific leaf area than GO_NA plants (upper‐case letters, Figure 3c). Notably, there was no correlation between the specific leaf area of JE_IN plants (measured under 2:1 growth condition, data not shown) at the first harvest and the length of the longest leaf (Figure S1h).

For the carbon to nitrogen ratio, there was overall no significant difference between populations at the first and the second harvest (Figure S1g, Table 1). However, there were significant differences between the replacement series for the carbon to nitrogen ratio at the first and second harvest for JE_IN as target but not for GO_NA (lower‐case letters, Figure 4a,b, Table 2). For the pairwise comparison, there were no significant differences in carbon to nitrogen ratio between populations in intra‐ and interpopulation competition treatments (Figure 4a,b).

The water content of the leaves was overall different between populations at the first harvest, with JE_IN plants having a higher water content than GO_NA plants when grown in competition of two individuals (Figure S1e, Table 1). In addition, JE plants had a higher water content than GO_NA plants at the second harvest when grown in competition of three plants. Moreover, when JE_IN plants were targets in the replacement series, there was a significant difference for water content only at the first harvest between plants grown in interpopulation (1:2) and intrapopulation (0:2 and 0:3) competition, with plants under the latter conditions having a higher water content. In addition, JE_IN plants had a higher water content than GO_NA plants when grown in interpopulation (2:1) and intrapopulation competition (0:2) at the first harvest, and when grown in interpopulation competition (1:1 and 2:1) at the second harvest (lower‐case letters, Figure 4c,d).

**FIGURE 5:**
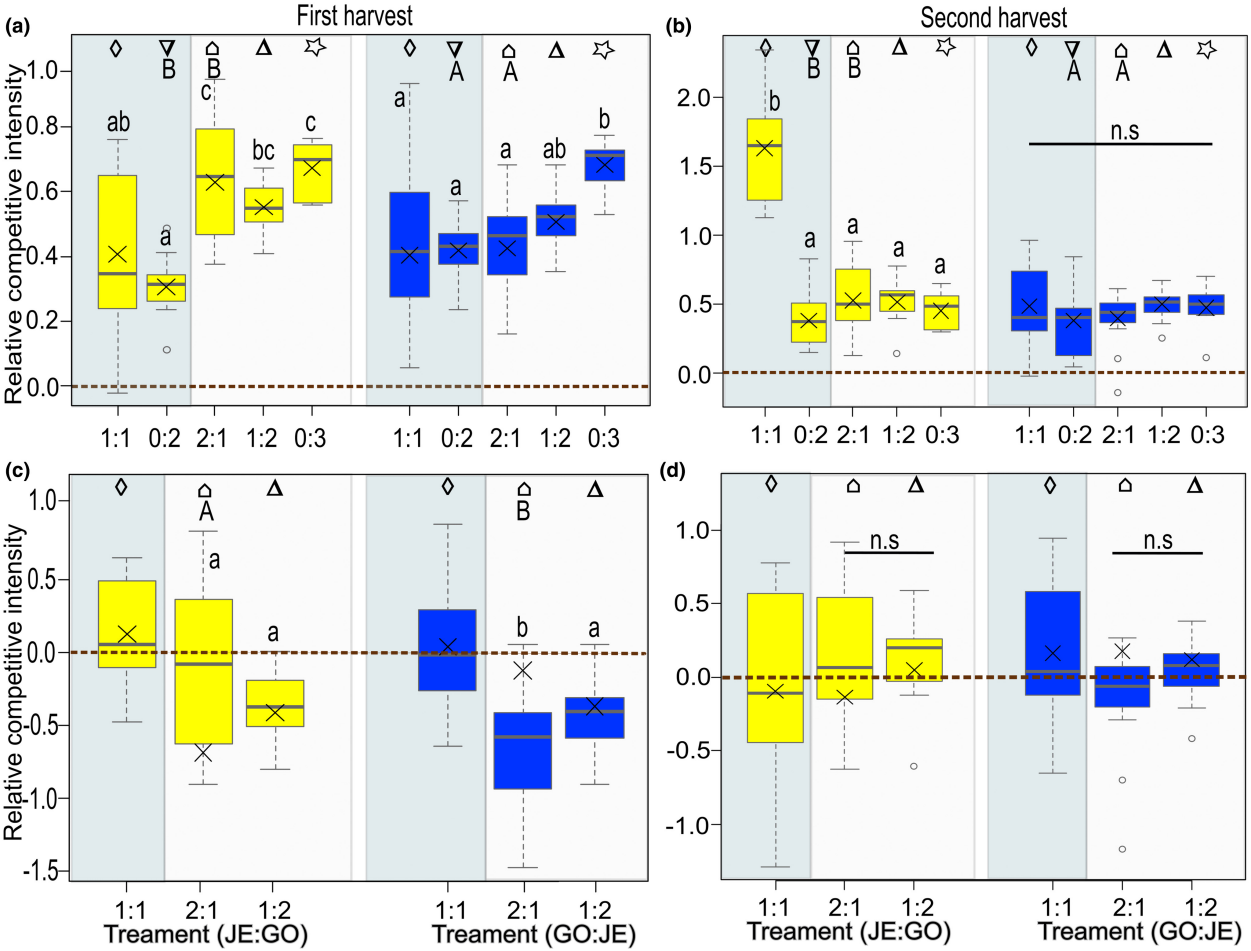
Competition traits of *Bunias orientalis* from plants of the naturalized population GO (yellow) and plants of the invasive population JE (blue), represented by (a, b) RCI relative to performance of control plants at the first harvest (artificial defoliation; a) and second harvest (after regrowth; b) and (c, d) RCI relative to performance of intrapopulation plants at the first (c) and second (d) harvest when grown in a replacement series: in interpopulation competition or in intrapopulation competition (number competitor: number target). Description of box and whisker symbols follow Figure 2, *n* = 13–15 for replacement series. The shaded box represent the competition treatments (*n* = 29–30 for competition of two and *n* = 36–37 for competition of three). Dashed lines depict the RCI equal to 0 (no competitive impact), positive values indicate a competitive impact of the competitor on the target individual, whereas negative values indicate the opposite. Letters indicate significant differences between replacement series (lower‐case letters) according to Tukey posthoc tests, n.s. indicates no significant difference (*p* > .05). Identical symbols indicate pairwise comparisons between plants of both populations grown under similar conditions, with different capital letters representing a significant difference between populations (*p* ≤ .05).

The overall RCI, relative to the performance of control plants, differed at the first harvest when plants were grown in competition of three plants and at the second harvest when they were grown in competition of two. Specifically, GO_NA plants showed a higher value, indicating a high competitive impact of JE_IN on GO_NA (Figure S2a, Table 1). Within the replacement series, the RCI differed for GO_NA targets, with a lower value when grown in intrapopulation competition (0:2) compared to competition with in total three plants (2:1, 1:2 and 0:3) at the first harvest (lower‐case letters, Figure 5b, Table 2). In addition, JE_IN plants as targets differed significantly, with a lower competitive ability when grown in inter‐ and intrapopulation competition (1:1, 0:2, and 2:1) compared to intrapopulation competition (0:3). For the second harvest, GO_NA plants as targets had significantly higher values for plants grown in 1:1 than under other growth conditions at the second harvest (lower‐case letters, Figure 5b, Table 2). In contrast, the RCI did not differ between JE_IN targets in the different replacement series. The overall RCI relative to the performance of plants grown in intrapopulation competition (0:2) did not differ between populations at both harvests (Figure S2c, Table 1). However, the overall RCI relative to the performance of plants grown in intrapopulation competition of three (0:3) differed at the first harvest and at the second harvest, with GO_NA plants showing a higher value than JE_IN plants (Figure S2c,d, Table 1). Within the replacement series, the RCI relative to the performance of plants grown in intrapopulation competition (0:3) only differed for JE_IN targets at the first but not at the second harvest, with a lower value under 1:2 than under 2:1 conditions (lower‐case letters, Figure 5c). However, no significant differences in competitive intensity were found for GO_NA targets in the different replacement series both at the first and at the second harvest relative to the performance of plants grown in intrapopulation competition (0:2 and 0:3) (Figure 5c,d, Table 2).

**FIGURE 6:**
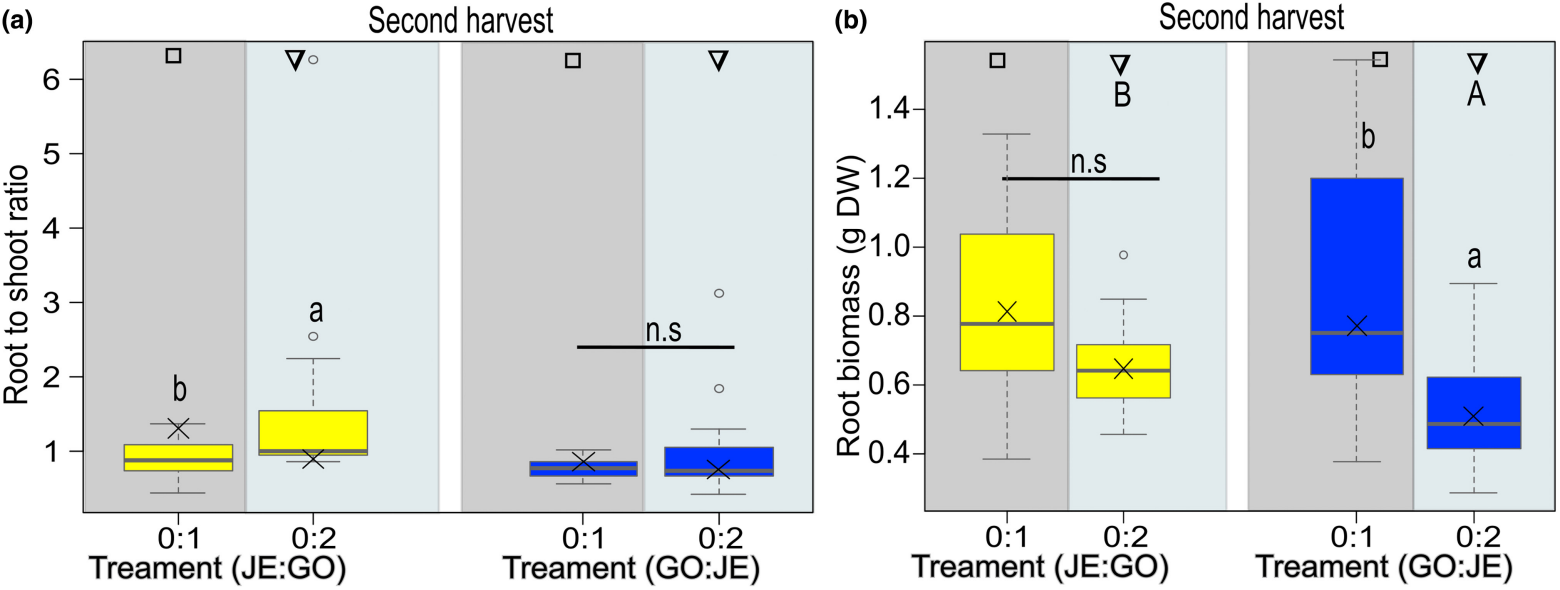
Root‐shoot biomass (a) and root biomass (b) of *Bunias orientalis* plants of the naturalized population GO (yellow) and plants of the invasive population JE (blue) when grown alone or in intrapopulation competition. Description of box and whisker symbols follow Figure 2, *n* = 14–15 for replacement series. The shaded boxes represent the competition treatments (control vs. intrapopulation competition of two). Letters indicate significant differences between replacement series (lower‐case letters) according to Tukey posthoc tests, n.s. indicates no significant difference (*p* > .05). Identical symbols indicate pairwise comparisons between plants of both populations grown under similar conditions, with different capital letters representing a significant difference between populations (*p* ≤ .05).

At the end of the experiment, the root to shoot ratio was measured from plants grown alone (0:1) or in intrapopulation competition (0:2). Overall, there was no significant difference between populations (Table 1). GO_NA plants showed a significantly lower root to shoot ratio when grown in intrapopulation (0:2) compared to GO_NA plants grown alone (0:1), while the root to shoot ratio of control plants and plants grown in intrapopulation competition did not differ for JE plants. There were also no differences in root to shoot ratio between populations in the pairwise comparisons (0:1 vs. 0:1and 0:2 vs. 0:2) (Figure 6a, Table 2). For root biomass, the only significant difference was found between populations when grown in intrapopulation competition (0:2), with GO_NA plants having a higher root biomass than JE_IN plants (upper‐case letters, Figure 6b, Table 1). However, the root biomass of GO_NA plants did not differ between the individuals grown in different replacement series, while JE_IN plants significantly differed, with control plants (0:1) having a higher root biomass than those grown under intrapopulation competition 0:2 (lower‐case letters, Figure 6b, Table 2).

## 4 DISCUSSION

This study showed that the performance and competitive intensity between *B. orientalis* plants from two populations, one of naturalized (GO) and one of invasive status (JE), differed in dependence of whether plants were grown alone or in intra‐ or interpopulation competition. Several of these differences may be assigned to the population status. Previous research on *B. orientalis* has revealed differences in various traits such as leaf number between native, naturalized and invasive populations, suggesting that population status can indeed shape plant characteristics (Binama & Müller, 2022; Tewes & Müller, 2018). While the observed differences in growth and competitive ability may be partially attributed to other factors, such as genetic history and different original environments, co‐varying between the populations, the high level of intraspecific trait variation found in the present study implies a prominent role of the invasion history in driving the plants' success. This finding aligns with prior research on invasive plant evolution, which highlights the potential for introduced populations to undergo rapid trait shifts in response to novel environments (Szűcs et al., 2017). Conducting our study under controlled conditions with F1 generations minimized the influence of external factors such as environmental variation and reduced maternal effects, respectively, strengthening the argument that the observed differences are likely due to intrinsic traits potentially resulting from different invasion history.

Competition, both within and between species, is among the most important biotic factors influencing plant growth and physiology (Luong & Loik, 2022; Song et al., 2017). In partial agreement, in our study competition consistently led to a reduction in traits related to growth and morphology, including aboveground biomass, total number of leaves and length of the longest leaf, i.e. plant expansion. However, traits related to physiology, such as nitrogen, carbon and water content, were less impacted in both populations. These data suggest that competition negatively affected those growth‐related traits that may characterize the ability of *B. orientalis* to grow and finally also reproduce and survive in their introduced ranges. However, further research employing targeted manipulations of specific traits is necessary to definitively isolate and quantify their individual contributions to competitive ability.

Averaged over all plants within a population, aboveground biomass did not differ between the GO_NA and JE_IN population at the first harvest. In contrast, after regrowth at the second harvest, JE_IN plants had overall a higher aboveground biomass than GO_NA plants when grown in competition of three individuals. Furthermore, when grown with two competitors from the GO_NA population, JE_IN plants had a higher aboveground biomass at both harvests, suggesting that, as the number of competitors increased, JE_NA plants invested more in aboveground biomass. Results from previous research have been inconclusive and solely focused on competition between invasive and native populations. In many species, plants of invasive populations have been shown to produce a higher aboveground biomass than plants of native populations (Beaton et al., 2011; Huang et al., 2012; Lin et al., 2015, 2018). However, the opposite pattern was also found (Bossdorf et al., 2004; Felker‐Quinn et al., 2013; Vilà et al., 2003). Not surprisingly, plants of *B. orientalis* produced less aboveground biomass when grown in competition than when grown alone in the present study, regardless of population origin, as may be expected when resources are limited. Within the replacement series, plant individuals grown in competition of three showed mixed values (low, high or no difference to plants grown alone and in competition of two), despite receiving more nutrients than control plants or those with two competitors. This suggests that nutrient limitation alone might not fully explain the observed patterns. However, potential confounding effects from different fertilization levels cannot be entirely excluded. Additionally, differences in the aboveground biomass in the replacement series between the GO_NA and the JE_IN targets suggests that the growth of GO_NA plants is negatively impacted by an increasing number of competitors regardless of their origins. In contrast, growth of JE_IN plants may be more negatively influenced by more kin competitors. Thus, whether intrapopulation competition has less negative impacts than interpopulation competition seems to depend on the population origin and this assumption cannot be generalized. In any case, our results indicate both neighbor‐ and density‐specific effects. Similarly, neighbor effects of invasive populations suppressing the biomass of native populations were found in other species (Beaton et al., 2011; Huang et al., 2012). Kin selection may be involved, suggesting that competition between related neighbors is lower than competition between nonrelatives (File et al., 2012; Platt & Bever, 2009). In addition, kin selection is likely density‐dependent, with competition between few related neighbors being lower than competition between larger groups of relatives. Yet, previous studies on kin recognition in plants have reported contrasting results (Lee et al., 2021; Lepik et al., 2012; Zhang et al., 2019).

The main assumption of the EICA hypothesis is that escape from specialist herbivores allows an evolutionary shift in resource allocation from defense to growth, resulting in invasive plants with higher competitive ability (Blossey & Notzold, 1995). In contrast to biomass values, GO_NA plants produced more leaves when grown in competition of two plants than JE_IN plants at the second harvest. However, in a competition of three, JE_IN plants had longer leaves than GO_NA plants after both harvests. These results suggest that plants from the GO_NA and JE_IN population allocate their resources particularly into leaf expansion when grown in competition to utilize light resources effectively and potentially limit light for competitors. Under conditions of reduced light availability, plants may allocate more biomass to the shoots, produce larger leaves and enhance the elongation of stems (Weiner, 1993; Westoby et al., 2002). Such larger vegetative expansion has also been found in invasive populations of other species in both competition and competition‐free studies (Chun et al., 2010; Lee et al., 2021). As for aboveground biomass, the number and length of leaves of *B. orientalis* were reduced when plants were grown in competition, indicating that competition can reduce fitness‐related traits (Craine & Dybzinski, 2013; Harper, 1977).

Specific leaf area (SLA) is known to be a key functional trait that can relate to leaf length and light capture (Pérez‐Harguindeguy et al., 2013). Across all *B. orientalis* samples, leaves of the JE_IN plants had a higher SLA than those of the GO_NA plants. However, no significant relationship was found between length of the longest leaves and SLA in JE_IN plants. Plant might adopt a strategy of allocating resources more efficiently to individual leaves. This could result in a higher SLA to enhance the efficiency of light capture (Liu et al., 2016). In addition, JE_IN plants had a higher SLA when grown in intrapopulation than in interpopulation competition or grown alone. These responses are obviously species‐specific; in contrast to the findings in *B. orientalis*, SLA did not differ between invasive *Taraxacum officinale* grown in intrapopulation competition or alone (Lee et al., 2021).

The overall leaf carbon to nitrogen ratio was comparable between the two *B. orientalis* populations at both harvests. However, JE_IN targets grown in competition had a higher carbon to nitrogen ratio than control plants. In grasses and sedges, a higher carbon to nitrogen ratio was found to be associated with a higher nitrogen use efficiency and thus, a higher competitive ability (Peng et al., 2023). The water content was mostly higher in JE_IN plants compared to GO_NA plants of *B. orientalis* when grown in competition. The availability of nitrogen and water resources frequently limits plant growth (Huntley, 2023; Thomas & Ougham, 2015). While both populations used partly similar, partly different resource allocation strategies, plants of the invasive JE_IN population may have the ability to more successfully exploit these resources, resulting in a competitive advantage. The higher competitive ability of JE_IN than GO_NA plants may be associated with their greater resource allocation in more and longer leaves as well as an increased biomass production, at least when plants were grown in competition of three individuals. An increase of plant density enhances the level of competition, which may occur for space, nutrients, and/or light availability (Daehler, 2003; Davis, 2009). Our results are in partial agreement to previous studies, which have associated the competitive superiority of invasive over native populations with shifts in biomass allocation rather than increased individual size (Meyer & Hull‐Sanders, 2008; Te Beest et al., 2009; Zheng et al., 2015). We here provided initial observations of potential trait differences between naturalized and invasive populations of *B. orientalis*. However, the limited sample size (one population per status) does not allow to isolate these differences as sole drivers of invasive plant competitiveness. To strengthen the hypothesis, further research is needed.

After defoliation and regrowth, i.e. at the second harvest, most of the plant traits that did not differ between populations when grown in competition of two or three plants at the first harvest were significantly different, with the exception of physiological traits. An increased allocation of resources towards shoots is indicative of a high level of competitive interactions between aboveground plant structures, whereas a high allocation of resources towards roots suggests a dominance of competitive interactions between roots (Berendse & Möller, 2009; Janeček et al., 2014). The findings of this study did not show a significant difference of root to shoot ratio between populations after regrowth. However, the root to shoot ratio of GO_NA plants decreased with intrapopulation competition compared to control plants. An increased aboveground biomass of GO‐NA plants in intrapopulation competition compared to JE_IN plants at the first but not the second harvest may suggest a trade‐off that occurs as a result of resource allocation mostly into aboveground biomass. In contrast, a lower biomass allocation to roots in an invasive population compared to plants from a native population at regrowth has been previously found in *J. vulgaris* and may be explained by the fact that some invasive plants trade their regrowth ability for better growth performance (Lin et al., 2018). With a high root to shoot ratio, individuals may contain more storage resources in the roots for regrowth (Chen et al., 2013; McCormick et al., 2013). Several plant species exhibit alterations in their root to shoot ratio when exposed to shading or limited nutrient supply (Brouwer, 1965; Qi et al., 2019). Similar as for the aboveground biomass, GO_NA plants grown in intrapopulation competition had a higher root biomass than JE_IN plants. These findings could imply that GO‐NA plants respond similarly to intrapopulation competition of two plants in terms of investment in aboveground versus belowground biomass, revealing a comparable strategy for reducing competition with kin neighbors.

In summary, the results of our study show that plants of the invasive JE population outperformed the naturalized GO population and had a higher competitive ability than the naturalized population in herbivore‐free conditions. However, the outcome may differ in presence of herbivores and may depend on the type or level of herbivory, as shown in *J. vulgaris* (Lin et al., 2015). The observed differences in growth traits between the naturalized and the invasive population could be attributed to pre‐adaptation and post‐introduction evolutionary processes occurring in the invasive range (Tewes & Müller, 2018). Invasive populations are often released from natural enemies, which could lead to the reallocation of resources from defense mechanisms to growth and competitive ability, as postulated by the EICA hypothesis (Blossey & Notzold, 1995). More research is needed comparing competitive abilities of populations along the full introduction‐naturalization‐invasion continuum and studies should consider also competition after instances of defoliation, as done in the present study.

## AUTHOR CONTRIBUTIONS

**Blaise Binama:** Conceptualization (equal); data curation (equal); formal analysis (lead); investigation (lead); methodology (equal); writing – original draft (lead). **Müller Caroline:** Conceptualization (equal); data curation (equal); investigation (equal); project administration (lead); writing – review and editing (lead).

## CONFLICT OF INTEREST STATEMENT

The authors declare that there are no competing interests.

## Supporting information


Figure S1. and S2.

Table S1.

## Data Availability

The raw data and code can be found in https://github.com/bbinama/Growth‐and‐competition‐between‐plants‐of‐a‐naturalized‐and‐an‐invasive‐population‐of‐Bunias‐oriental.git.
