# Supplementary material for: Differences in growth and competition between plants of a naturalized and an invasive population of Bunias orientalis
Source: Ecol Evol. 2024 Mar 18;14(3):e11153. doi: 10.1002/ece3.11153 (PMC10948592; doi:10.1002/ece3.11153)
Supplement: Supplementary file 1 — Figure S1. and S2. [file ECE3-14-e11153-s001.pdf]

Supplement for: Differences in growth and competitive ability between a naturalized and an invasive population of *Bunias orientalis*

Blaise Binama[1], Caroline Müller[1*]

[1] Department of Chemical Ecology, Bielefeld University, Universitätsstr. 24, 33615 Bielefeld, Germany

* Corresponding author. E-mail: caroline.mueller@uni-bielefeld.de

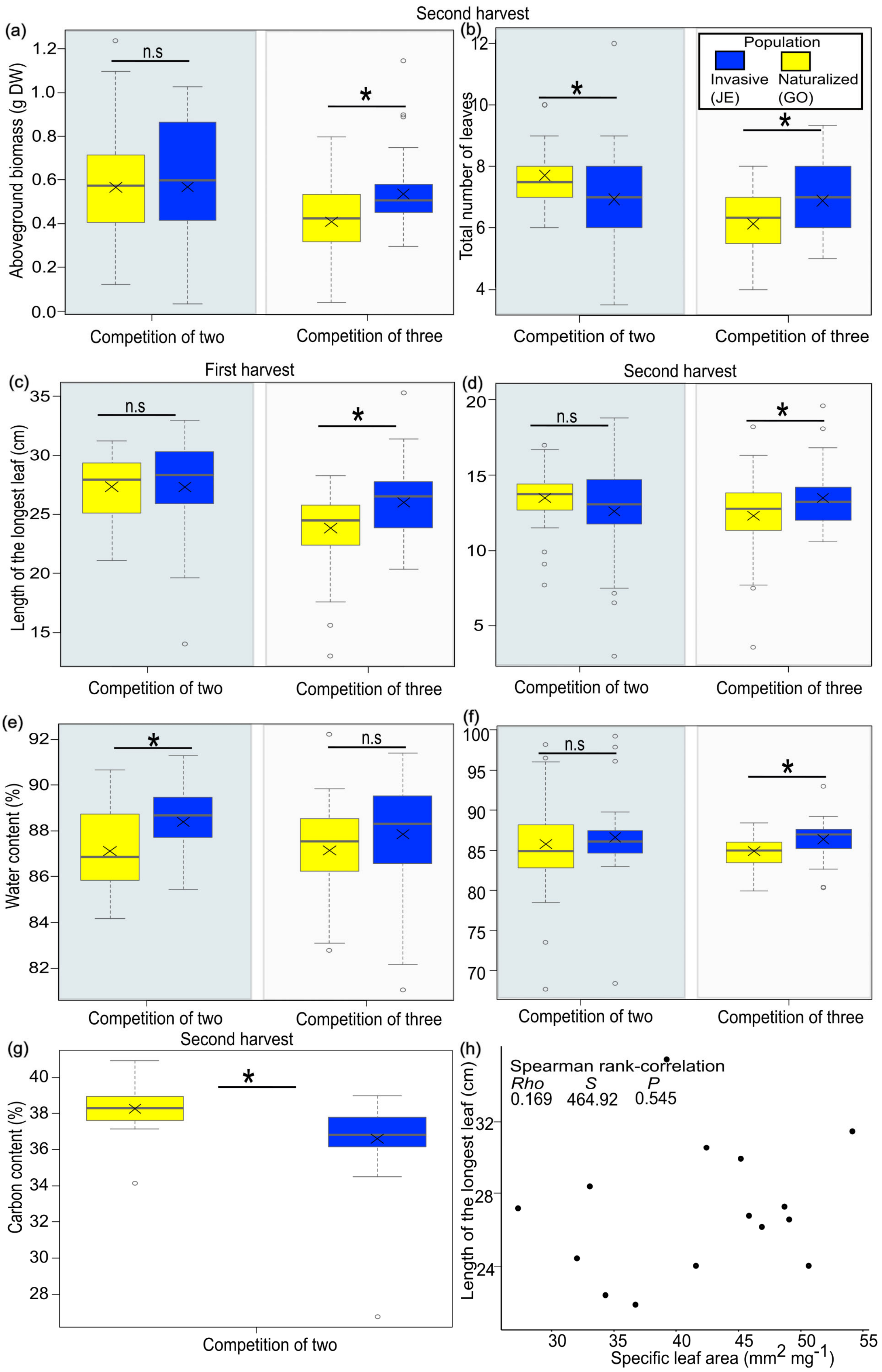

Second harvest
(a)
Aboveground biomass (g DW)
n.s
*
Competition of two
Competition of three
(b)
Total number of leaves
Population
Invasive (JE)
Naturalized (GO)
*
*
Competition of two
Competition of three
(c)
First harvest
Length of the longest leaf (cm)
n.s
*
Competition of two
Competition of three
(d)
Second harvest
n.s
*
Competition of two
Competition of three
(e)
Water content (%)
*
n.s
Competition of two
Competition of three
(f)
n.s
*
Competition of two
Competition of three
(g)
Second harvest
Carbon content (%)
*
Competition of two
(h)
Spearman rank-correlation
Rho S P
0.169 464.92 0.545
Length of the longest leaf (cm)
Specific leaf area (mm2 mg-1)

Figure S1. Overall growth and physiological traits of *Bunias orientalis* from plants of the naturalized population GO (yellow) and plants of the invasive population JE (blue), represented by (a) aboveground biomass at the second harvest, b) total number of leaves at the second harvest, (c, d) length of the longest leaf at the first harvest (artificial defoliation; c) and second harvest (after regrowth; (d), (e, f) water content at the first (e) and second (f) harvest, (g) carbon content at the second harvest when grown in a competition of two plants or competition of three plants, (h) correlation between length of the longest leaf and specific leaf area in leaves of *Bunias orientalis* grown in replacement series 2:1 at first harvest; rho: coefficient of determination (Spearman rank correlation). Data are presented as box whisker plots, boxes represent the 25th and 75th percentiles and medians, crosses show means, whiskers mark minimum and maximum within 1.5-fold interquartile ranges and open dots are outliers. The shaded boxes represent the competition treatments (n = 29-30 for competition of two and n = 36-37 for competition of three); n.s. indicates no significant difference ($p > 0.05$), * indicates significant differences between populations ($p \leq 0.05$).

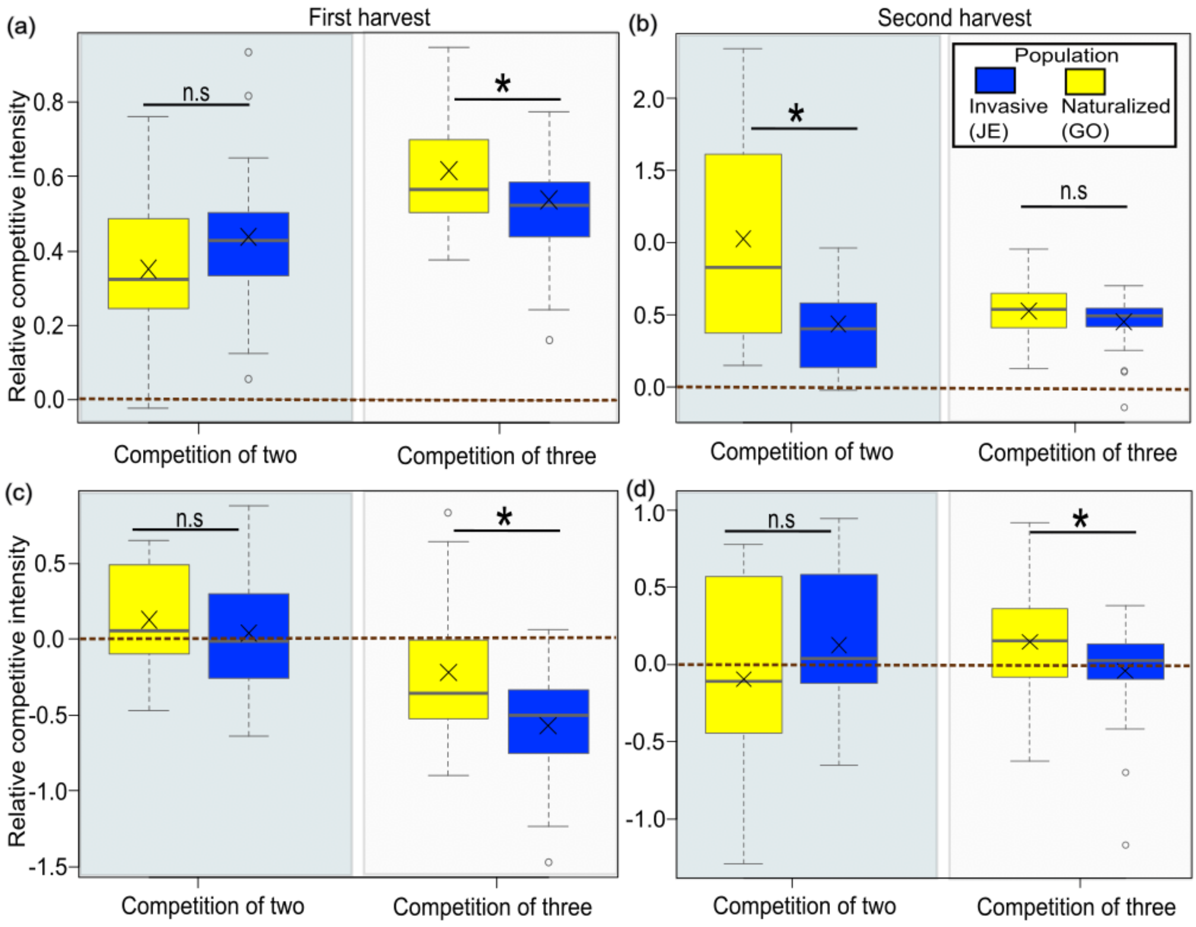

Figure S2. Overall relative competitive intensity of *Bunias orientalis* from plants of the naturalized population GO (yellow) and plants of the invasive population JE (blue), represented by (a, b) relative competitive intensity relative to performance of control at the first harvest (artificial defoliation; a) and second harvest (after regrowth; b), and (c, d) relative competitive intensity relative to performance of intrapopulation competition (0:2 or 0:3) at the first (c) and second (d) harvest when grown in a competition of two plants or competition of three plants. Description of box and whisker symbols as in Figure S1. The shaded boxes represent the competition treatments (n = 29-30 for competition of two and n = 36-37 for competition of three); n.s. indicates no significant difference ($p > 0.05$), * indicates significant differences between populations ($p \leq 0.05$).
